# Enabling a novel solvent method on Albendazole solid dispersion to improve the in vivo bioavailability

**DOI:** 10.1016/j.ejps.2024.106751

**Published:** 2024-05-01

**Authors:** Ming-Jie Han, Zhiyang Zack Zou

**Affiliations:** Department of DMPK, Global Health Drug Discovery Institute, Zhongguancun Dongsheng International Science Park, Beijing, PR China

**Keywords:** Albendazole, Solid dispersion, Dissolution rate, Bioavailability, Solvent method

## Abstract

Albendazole, a vital medication endorsed by the World Health Organization for combating parasitic infections, encounters a challenge stemming from its low solubility, significantly impeding absorption and bioavailability. Albendazole has near-insolubility in most organic solvents, so the solid dispersions of albendazole were predominantly using the fusion method. However, the solvent method could offer the advantage of achieving molecular-level mixing homogeneity. In this investigation, we incorporated the pH adjustment to prepare albendazole solid dispersion using a solvent method, which utilizes trace amounts of HCl in methanol, yielding notably enhanced albendazole solubility. Subsequently, carriers such as PEG6000/Poloxamer 188 (PEG: polyethylene glycol) and PVP K30/Poloxamer 188 (PVP: polyvinylpyrrolidone) were employed to create albendazole solid dispersions. Comprehensive characterization through dissolution rate analysis, PXRD (Powder X-ray diffraction), SEM (Scanning electron microscopy), DSC (differential scanning calorimetry), and pharmacokinetic (PK) studies in mice and rats was conducted. The findings indicate that the solid dispersion effectively transforms the crystalline state of albendazole into an amorphous state, resulting in significantly enhanced in vivo absorption and a 5.9-fold increase in exposure. Besides, the exposure increased 1.64 times of commercial albendazole tablets. Notably, PEG6000/Poloxamer 188 and PVP K30/Poloxamer 188 solid dispersions exhibited superior dissolution rates and pharmacokinetic profiles compared to commercially available albendazole tablets.

## Graphical abstract

**Figure:**
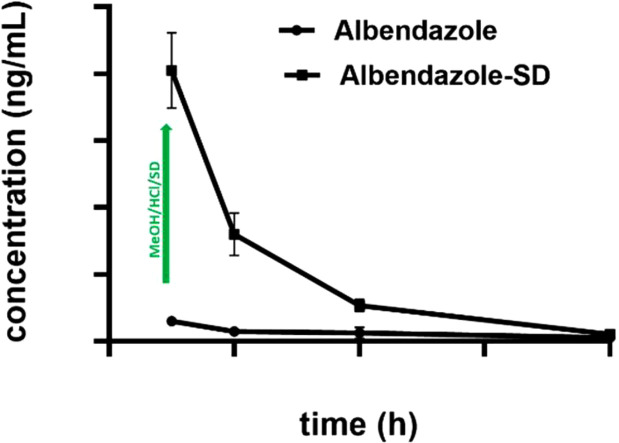

Albendazole, a vital medication endorsed by the World Health Organization for combating parasitic infections, encounters a challenge stemming from its low solubility, significantly impeding the absorption and bioavailability. In this investigation, we engaged the pH adjustment to prepare albendazole solid dispersion using a solvent method, which utilizing trace amounts of HCl in methanol, yielding notably enhanced albendazole solubility. Subsequently, carriers such as PEG6000/Poloxamer 188 and PVP K30/Poloxamer 188 were employed to create albendazole solid dispersions. The solid dispersion effectively transforms the crystalline state of albendazole into an amorphous state, resulting in significantly enhanced in vivo absorption and a 5.9-fold increase in exposure. Besides, the exposure increased 1.64 times of commercial albendazole tablets. The albendazole solid dispersion using PEG6000/Poloxamer 188 and PVP K30/Poloxamer 188 as carrier through solvent method exhibited superior dissolution rates and pharmacokinetic profiles compared to commercially available albendazole tablets.

**Image for graphical abstract**

## 1 Introduction

Albendazole (methyl [5-(propylthio)−1H-benzimidazol-2-yl] carbamate) is a broad-spectrum anthelmintic drug that belongs to the benzimidazole class (Bolla and Nangia, 2018; Scott et al., 2020). Albendazole interferes with the parasite's ability to absorb glucose, leading to a depletion of glycogen stores (Shahriar and Alpern, 2020). It also disrupts microtubule formation in the parasite cells, inhibiting their ability to divide and reproduce (Riches et al., 2020). These actions contribute to the elimination of parasitic infections (Hotez, 2017). It is widely used in the treatment of various parasitic-infected human diseases such as hydatidosis, ascariasis, neurocysticercosis, Hydatid disease, trichuriasis, and so on (Malik and Dua, 2023). Albendazole is listed on the World Health Organization's List of Essential Medicines, emphasizing its importance in global healthcare (Malik and Dua, 2023). It is commonly used in mass drug administration programs in regions where parasitic infections are endemic.

Albendazole, classified as a type two drug, exhibits poor solubility, leading to suboptimal bioavailability and impacting its therapeutic efficacy. Albendazole is a fundamentally lipophilic compound with a logD of 3.47 at pH7 and a pKa of 10.43 (Meena et al., 2012; Swamy and Basavaiah, 2014). To address these challenges, various formulation strategies have been explored to enhance its aqueous solubility (Kalaiselvan et al., 2006; Suzuki et al., 2021; Gradzielski et al., 2021). These include incorporating surfactants like Tween 80 and bile salts (Eriksen et al., 2022), developing self-micro emulsification formulations (Meena et al., 2012), solid lipid nanoparticles (Kumar et al., 2020), and co-crystals. Among these, solid dispersion emerges as the most effective approach. The mechanism underlying increased solubility involves the hydrophilic carrier enhancing the drug dissolution rate by reducing crystal size and particle porosity. Solid dispersions are categorized into eutectic systems, glass solutions, suspensions, and solid solutions based on molecular arrangement (Malkawi et al., 2022; van Hoogevest, 2017; Mai et al., 2020). Various technologies, including the hot-melt extrusion method, solvent method, electro-spinning, and spray drying, have been employed for solid dispersion preparation (Williams et al., 2013). Numerous albendazole solid dispersions have been created using methods such as hot-melt extrusion with PVP K12 as the carrier (Martinez-Marcos et al., 2016), kneading and solvent evaporation with HPMC (Hydroxypropyl Methyl Cellulose) as the carrier, fusion method with Gelucire 50/13 and PEG15000 (Suzuki et al., 2021), and fusion method with PEG6000 and Poloxamer188 (Dong et al., 2020). However, certain methods encounter challenges like low drug loading capacity and slow dissolution rates. Hence, there is a pressing need for further advancements in albendazole solid dispersion techniques to overcome these limitations.

**Scheme 1:**
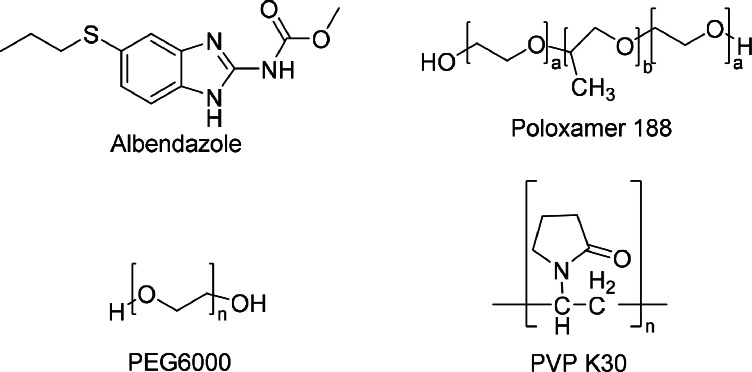
The chemical structure of albendazole, Poloxamer 188, PEG6000, and PVP K30.

The solvent method for solid dispersion preparation holds the potential for achieving molecular-level mixing homogeneity, thereby substantially enhancing the properties of low-solubility compounds (Bai et al., 2011; Almotairy et al., 2021; Yang et al., 2014). The microenvironmental pH (pH M) represents the solution pH surrounding the drug particles, and pH adjustment emerges as a key strategy for improving the solubility of poorly soluble drugs (Bai et al., 2011). Particularly, weakly basic drugs with limited solubility can experience enhanced solubilization in an acidic pH environment, such as telmisartan (Almotairy et al., 2021) and toltrazuril (Zhang et al., 2019) . Albendazole, known for its challenging solubility in organic solvents, presented a significant hurdle for the solvent method. Given Albendazole's weak basic nitrogen, we introduced an acidic modification to the solvent, aiming to ameliorate its solubility. In this investigation, pH adjustment played a pivotal role in the preparation of albendazole solid dispersion via the solvent method. Trace amounts of HCl in methanol were utilized, resulting in a notable improvement in albendazole solubility. We innovatively formulated a novel albendazole solid dispersion using the solvent method, employing PEG600, PVP, and Poloxamer 188 as carriers. Comprehensive characterization of the solid dispersion was conducted through assessments of dissolution rate, PXRD, DSC, SEM, and PK exposure.

## 2 Materials and methods

Albendazole was procured from Bidepharm Company, while PEG6000 was obtained from Thermo-scientific Company. Poloxamer 188 was sourced from Rhawn Company, and PVP K30 was acquired from TCI Company. The simulated gastric fluid (SGF) utilized in the experiment was supplied by MesGen Biotechnology. The albendazole tablet was from Tianjin Smith Kline &French Laboratorles Ltd. All additional reagents and chemicals employed for analysis and dilution purposes were of analytical grade.

### 2.1 Preparation of Albendazole solid dispersion

Albendazole solid dispersion was prepared using the solvent method: Initially, Albendazole (10 mg) was dissolved in 4 mL of MeOH, and the resulting suspension was clarified upon the addition of 2 µL HCl. Carriers (40 mg) with varying polymer ratios were dissolved in MeOH (6 mM) through either vortex mixing or ultrasonication. Subsequently, the Albendazole solution was introduced into the carrier solution and vortexed for five minutes. The solvent was removed using a rotary evaporator. The resulting solid dispersion was ground and filtered through a 60-mesh sieve. A substantial quantity of solid dispersion was generated. The solid dispersion of albendazole could be scaled to gram or kilogram level using the spray dry method.

### 2.2 Phase solubility studies

An excess amount of albendazole was introduced into 10 mL of water, which contained varying ratios of PEG6000, poloxamer188, and PVP K30. The resulting mixture underwent agitation at 60 rpm for a period of three days. Subsequently, the dissolved albendazole was separated through filtration, and its absorption at 300 nm was monitored using a UV–Vis spectrophotometer (SpectraMax M5).

### 2.3 Dissolution study

Albendazole, Albendazole solid dispersion, and a physical mixture, each containing 20 mg of albendazole, were introduced into 200 ml of prewarmed simulated gastric fluid (SGF). The resulting mixture underwent agitation at 37 °C with a speed of 150 rpm. Samples were extracted at various time points, including 5 min, 10 min, 15 min, 20 min, 30 min, and 60 min, in aliquots of 200 µL. These samples were then filtered using a 0.22 µM syringe filter. Following filtration, 100 µL of each sample was combined with 100 µL of methanol and immediately vortexed to prevent new precipitation. Albendazole concentrations in the samples were subsequently determined using a UV–Vis spectrophotometer (M5 plate reader).

### 2.4 Powder X-Ray diffraction (PXRD)

PXRD data were collected on a Shimadzu X-ray Diffractometer XRD-7000. The X-ray tube includes a normal focus of 2.2 kW with a focal spot 1.0 × 10 mm. The samples were scanned from 0 to 50 with an acquisition time of 0.1 s.

### 2.5 PK study

The pharmacokinetic studies of Albendazole and its solid dispersion were conducted on BALB/c mice and SD rat orally, adhering to rigorous animal husbandry protocols. The procedures in this study were in strict compliance with the Animal Welfare Act, the National Research Council Guide for the Care and Use of Laboratory Animals (8th edition), and the National Laboratory Animal Management Regulations (2017). To ensure optimal conditions for testing, the animals underwent an overnight fasting period before administration. Each experimental group comprised three male SD rats or mice from Charles River Laboratories. Albendazole and its solid dispersion were suspended in PBS and administered to the mice and rat orally, with each dose of albendazole set at 10 mL/kg. The drug concentration of formulation was identified by LC-MSMS and all the doses were administrated to mice and rats totally. Blood samples were systematically collected at designated time intervals, specifically at 0.25 h, 0.5 h, 1 h, 2 h, 4 h, 8 h, and 24 h post-dosing. Subsequently, all blood samples underwent centrifugation at 1000 × *g* for 15 min and were then stored in *a* − 80 °C refrigerator to facilitate LC-MS/MS analysis. The PK studies were approved by IACUC with the approval number for mice is PK-M-07,182,023 on 2023–03–24, and the approval number for rat is PK-R-06,012,023 on 2023–03–24.

## 3 Results and discussion

As previously documented, numerous polymers have been identified for their potential to markedly enhance the solubility of albendazole, including PEG6000, Polyvinylpyrrolidone K30 (PVP K30), Poloxamer 188, Gelucire 50/13, cyclodextrin, and others. While a variety of solid dispersion formulations exist, many employ the fusion method and the hot-melt extrusion method, achieving a well-distributed drug-carrier blend, albeit with limited homogeneity. The solvent method, however, stands out for its ability to molecular-level to mix the drug into the carrier, yielding the most homogeneous solid dispersions (Leuner and Dressman, 2000). Albendazole, inherently insoluble in water and exhibiting minimal solubility in organic solvents such as methanol, ethanol, acetone, dichloromethane, and N, N-dimethylformamide, presents a challenge when attempting to scale up solid dispersion production using the solvent method. In this study, a novel approach was adopted wherein albendazole was suspended in methanol, followed by the addition of 0.5 ‰ concentrated HCl. This unique method resulted in a clear albendazole solution, facilitating facile mixing with other carriers to prepare solid dispersions. Notably, this technique allows for the scalable production of albendazole solid dispersions, overcoming the limitations associated with the use of pure organic solvents in the solvent method.

**Fig. 1:**
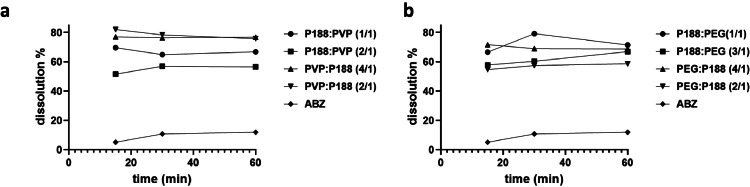
Screening the carrier ratios of albendazole solid dispersion. a) screening the ratio of poloxamer188 (P188) and PVP K30; b) screening the ratio of poloxamer188 (P188) and PEG6000. P188: Poloxamer188; PVP: PVP K30; ABZ: albendazole.

Firstly, we take the phase solubility study to investigate the solubilization of 5 % carriers for three days at 37 °C. The solubility of albendazole in PVP K30, PEG6000, Poloxamer188 are 22.5 µg/mL, 18.9 µg/mL, and 38.4 µg/mL respectively. However, when we use the combination of Poloxamer188/PVP K30 and Poloxamer188/PEG6000, the solubility of albendazole were significantly enhanced to 38.4 µg/mL and 31.4 µg/mL respectively. So we chose Poloxamer 188 in combination with PEG6000 and PVP K30 as carriers, respectively, and systematically screened their ratios, as illustrated in Fig. 1. Initially, we focused on the combination of Poloxamer 188 and PVP K30, preparing albendazole solid dispersions with ratios of 1:1, 2:1, 1:2, and 1:4. Dissolution rates were assessed in simulated gastric fluid, and sample analysis was conducted using absorption at 300 nm. Remarkably, all solid dispersions exhibited a significant enhancement in the solubility of pure albendazole. Particularly noteworthy was the PVP K30 and Poloxamer 188 (2:1) ratio, which achieved a dissolution rate exceeding 80 % for albendazole, in stark contrast to the less than 20 % dissolution rate observed for pure albendazole. Altering the ratio of PVP K30 or Poloxamer 188 resulted in a reduction in dissolution rate compared to the optimal PVP K30 and Poloxamer 188 (2:1) ratio. Simultaneously, we assessed the dissolution rates of solid dispersions using PEG6000 and Poloxamer 188 as carriers. This combination also demonstrated a significant increase in albendazole solubility. Notably, PEG6000 and Poloxamer 188 (1:1) exhibited the highest dissolution rate. Incrementing the content of either PEG6000 or Poloxamer 188 led to a decrease in dissolution rate. Furthermore, the dissolution rates of PEG6000/Poloxamer 188 (1:1) and PVP K30/Poloxamer 188 (2:1) combinations were found to be comparable. Consequently, we selected these two carrier combinations for the subsequent preparation of albendazole solid dispersions, warranting further investigation.

### 3.1 Dissolution study

**Fig. 2:**
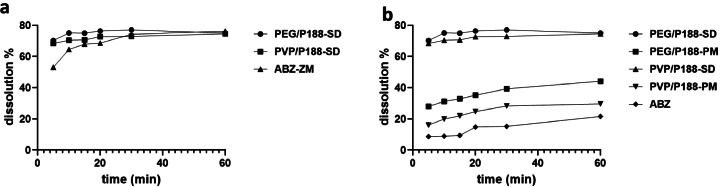
Comparison of dissolution rate of albendazole and its solid dispersion, physical mixture, and commercial tablet. a) Dissolution rate comparison between albendazole solid dispersion and albendazole tablet from Tianjin Smith Kline &French Laboratorles Ltd. b) Dissolution rate comparison between albendazole solid dispersion and their physical mixture. P188: Poloxamer188; PVP: PVP K30; ABZ: albendazole. ABZ-ZM: albendazole tablet from Tianjin Smith Kline &French Laboratorles Ltd.

The dissolution and release of a drug are pivotal factors influencing its absorption and bioavailability, thereby playing a crucial role in drug efficacy and toxicity (Raman et al., 2023; Nashed et al., 2023). In our investigation of albendazole, we focused on evaluating the dissolution rate of albendazole solid dispersion and its physical mixture in simulated gastric fluid. To prepare the samples, all materials underwent grinding and filtration through a 60-mesh filter, and the results are presented in Fig. 2. Notably, albendazole exhibited slow dissolution, with less than a 22 % release within 60 min and a dissolution rate below 28 % within 120 min. This characteristic can be attributed to albendazole's inherent low solubility (0.01 mg/mL) and poor wettability, as evidenced by a contact angle of 90 °C. The limited solubility and wettability of albendazole underscore the importance of addressing these challenges to enhance its overall solubility. Consequently, any approach aimed at promoting drug dispersion and increasing the available water surface could significantly contribute to improving albendazole solubility (Sahu and Kumar, 2022). By addressing these key characteristics, strategies can be devised to enhance the dissolution and release rates of albendazole, ultimately contributing to its therapeutic effectiveness and minimizing potential toxicity concerns.

The solid dispersion and physical mixture of albendazole exhibited accelerated dissolution rates compared to the pure drug, owing to the inclusion of carriers. In the case of albendazole physical mixtures, the dissolution rates were 44 % with PEG6000/Poloxamer188 and 30 % with PVP K30/Poloxamer 188 within 60 min, both surpassing the dissolution rate of pure albendazole. This observation implies that the two carrier combinations could substantially enhance the solubility of albendazole. Moreover, albendazole solid dispersion with PEG6000/Poloxamer188 (1:1) as a carrier demonstrated exceptionally rapid dissolution, exceeding 70 % within 5 min and reaching 77 % within 30 min. Similarly, when utilizing PVP K30/Poloxamer 188 as the carrier, the dissolution rate achieved 68 % within 5 min and 74 % within 1 hour. Comparatively, the PEG6000/Poloxamer188 carrier exhibited a higher capacity to improve albendazole solubility than PVP K30/Poloxamer 188, while the latter demonstrated better solidity. Consequently, both carrier combinations emerged as favorable choices for albendazole solid dispersion. The mechanism behind this enhancement in dissolution lies in the solubility properties of the carriers—PEG6000, Poloxamer188, and PVP K30. These carriers significantly improved the drug's wettability, reduced particle size, and prevented crystal aggregation, thereby elevating the dissolution rate of albendazole. The synergistic effects of these carriers contribute to the overall enhancement of albendazole solubility and dissolution characteristics.

Comparisons between the in-house prepared albendazole solid dispersion and its commercial counterpart yielded compelling findings. The solid dispersion formulations employing PEG6000/Poloxamer188 or PVP K30/Poloxamer188 as carriers exhibited notably faster dissolution rates than the commercial product. The commercial variant achieved only 53 % dissolution within 5 min and 65 % within 15 min, underscoring its comparatively slower performance. This discrepancy suggests that the solvent-based method employed in crafting albendazole solid dispersion using PEG6000/Poloxamer188 and PVP K30/Poloxamer188 carries distinct advantages over the commercial alternative. The in-house preparation method appears to offer a promising approach for enhancing drug dissolution rates, potentially leading to improved bioavailability and therapeutic outcomes. These findings emphasize the potential benefits of tailored formulations in optimizing the performance of albendazole and highlight the importance of the choice of carriers in the solid dispersion preparation process.

### 3.2 SEM microphotographs

**Fig. 3:**
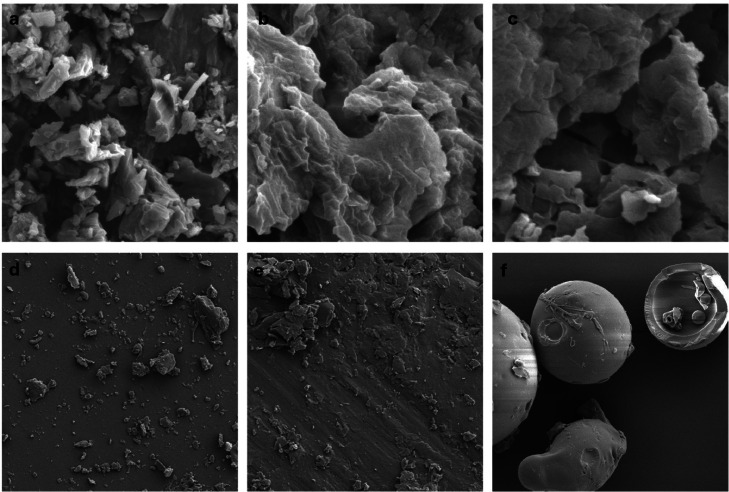
SEM microphotographs of a) Albendazole 2000x; b) Albendazole PEG600/Poloxamer188 solid dispersion, 2000x; c) Albendazole PVP K30/Poloxamer188 solid dispersion, 2000x; d) Poloxamer188, 400x; e) PEG6000, 400x; f) PVP K30, 400x**.**

To investigate the surface morphology of albendazole and its solid dispersion, the SEM microphotographs was employed. In Fig. 3a and 3b, the image of pure albendazole reveals crystalline particles. Contrastingly, the albendazole solid dispersion with PEG600/Poloxamer188 and PVP K30/Poloxamer188 exhibits irregular particles, presenting a rough and uneven surface. These images unequivocally confirm the transition of albendazole from its crystalline structure to an amorphous form. The SEM microphotographs vividly depict voluminous particles and distinct arrangements between the drug and carriers, providing insights into the amalgamation of albendazole into the polymer matrix.

### 3.3 PXRD study

The XRD diffractograms of albendazole, PEG6000, Poloxamer188, and albendazole solid dispersion are illustrated in Fig. 3. The albendazole spectra align with previous reports, exhibiting peaks at 11.6°, 13.0°, 17.8°, 22.0°, and 24.0° within a diffraction angle range from 0° to 50° These peaks indicate the presence of a crystalline form in pure albendazole. The PEG6000 carrier spectrum displays two prominent diffraction peaks at 19.2° and 23.7°, along with a smaller peak at 14.6° Similarly, Poloxamer188 exhibits two high diffraction peaks at 19.2° and 23.3° The XRD spectra of the PEG6000/Poloxamer188 solid dispersion of albendazole presented two prominent peaks at 19.2° and 23.7°, as well as a smaller peak at 14.6°, mirroring the PEG6000 carrier. Importantly, there are no peaks corresponding to albendazole, indicating a complete transformation into the amorphous state for the PEG6000/Poloxamer188 solid dispersion. Contrastingly, the XRD spectra of PVP K30 reveal an absence of peaks. In the case of the PVP K30/Poloxamer188 solid dispersion of albendazole, only two minor peaks emerge at the positions of Poloxamer188. These outcomes signify a comprehensive transition of albendazole from a crystalline to an amorphous state. In summary, the XRD spectra underscore the transformation of albendazole into an amorphous state in both PEG6000/Poloxamer188 and PVP K30/Poloxamer188 solid dispersions. As previous report, the oxygen atom of PEG6000 could bind to the oxygen atom of Poloxamer188 through hydrogen bond (Dong et al., 2020). This amorphization phenomenon aligns with the notable enhancement in the dissolution rate of albendazole observed in these solid dispersion formulations (Dong et al., 2020). The solid dispersion of albendazole is very stable within two months through PXRD detection.

**Fig. 4:**
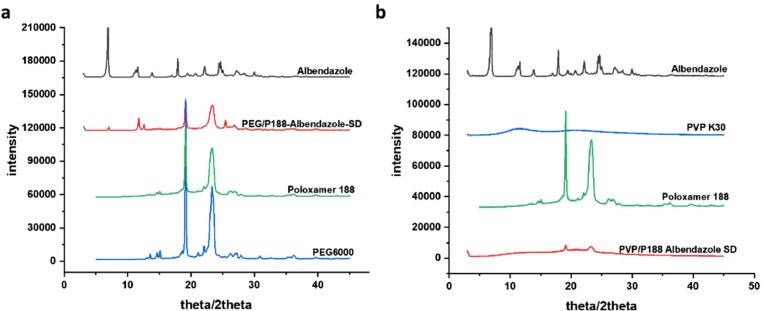
PXRD spectra of Albendazole and its solid dispersion. PVP: PVP K30; ABZ: albendazole; P188: Poloxamer188, PEG: PEG6000

### 3.4 DSC study

**Fig. 5:**
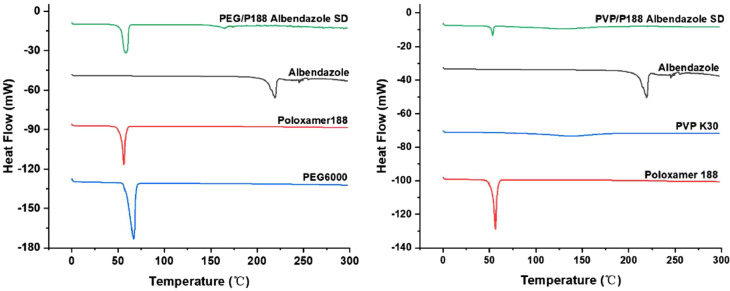
DSC thermograms of PEG6000, PVP K30, Poloxamer 188, Albendazole and its solid dispersions. P188: Poloxamer188, PEG: PEG6000, SD: solid dispersion.

The differential scanning calorimetry (DSC) is a widely employed thermal analysis method for investigating the physical properties of crystalline drugs. In this study, DSC analyses were conducted on PEG6000, PVP K30, Poloxamer 188, albendazole, and their respective solid dispersions using the NETZSCH High-Temperature Properties Series (Fig. 5). The thermal curves of PEG6000 and Poloxamer 188 exhibited distinct, sharp crystalline endothermic peaks at 64.0 °C and 56.3 °C, respectively. Conversely, PVP K30 did not display any discernible endothermic peak in its thermal curve. Albendazole exhibited a well-defined endothermic peak at 245.1 °C, indicative of its crystalline state. In the case of the PEG6000/Poloxamer 188 albendazole solid dispersion, the melting temperature remained at 64.0 °C, consistent with that of PEG6000. Notably, the albendazole melting peak at 245.1 °C disappeared, suggesting a complete transformation of albendazole from a crystalline to an amorphous state within the solid dispersion. Similarly, the PVP K30/Poloxamer188 albendazole solid dispersion's thermal curve exhibited a subtle peak at 64.0 °C, aligning with the melting temperature of PEG6000. This observation further supports the disappearance of albendazole's crystalline state within the solid dispersion. In summary, the DSC analyses underscore the efficacy of both PEG6000/Poloxamer 188 and PVP K30/Poloxamer188 solid dispersions in inducing the amorphization of albendazole, as evidenced by the elimination of its characteristic crystalline peaks in DSC.

### 3.5 Pharmacokinetics study

The in vivo pharmacokinetics of albendazole and its solid dispersions were explored in mice following oral administration, with the corresponding plasma concentration curves depicted in Fig. 5. Notably, the concentration of albendazole in plasma exhibited a substantial enhancement when administered in solid dispersion form. Specifically, the C_max_ of albendazole alone was measured at 15.07±3.3 ng/mL. In contrast, the C_max_ for albendazole in PEG6000/Poloxamer 188 solid dispersion reached 176.52±31 ng/mL, representing an 11.7-fold increase. Similarly, the C_max_ for albendazole in PVP K30/Poloxamer 188 solid dispersion was 202.54±28 ng/mL, marking a 13.4-fold augmentation compared to albendazole alone. This significant elevation in C_max_ implies an improved absorption and dissolution rate. Moreover, the AUC_last_ of albendazole alone was determined to be 19.82±6.9 ng*h/mL. In comparison, the AUC_last_ for albendazole in PEG6000/Poloxamer 188 solid dispersion was 108.73±21 ng*h/mL, reflecting a 5.49-fold increase. Similarly, the AUC_last_ for albendazole in PVP K30/Poloxamer 188 solid dispersion was 116.85±16 ng*h/mL, indicating a 5.90-fold enhancement. The heightened values of AUC and Cmax indicate that the solid dispersion of albendazole has the potential to enhance absorption and bioavailability, thereby elevating plasma concentrations. The Mean Residence Time (MRT) of albendazole was 4.03±2.25 h. In contrast, the MRT for albendazole solid dispersion was notably reduced to 1.39 h for PEG6000/Poloxamer 188 and 1.47 h for PVP K30/Poloxamer 188, respectively. This reduction in MRT suggests a significant increase in the absorption rate of albendazole, attributed to improvements in solubility and dissolution rate facilitated by the solid dispersion. These findings suggest that solid dispersion plays a crucial role, particularly in high doses, for enhancing the absorption of albendazole. The in vitro results further indicate that the dissolution rate of the solid dispersion correlates well with the in vivo absorption and bioavailability.

**Fig. 6:**
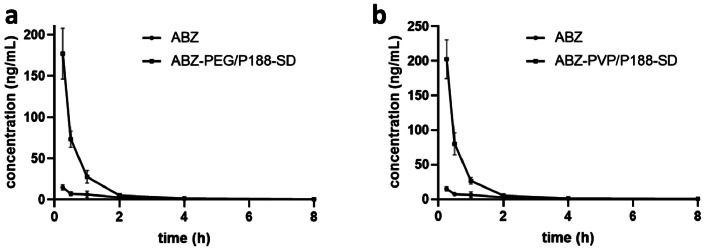
The plasma concentrations of albendazole after oral administration of albendazole and its solid dispersions to mice. a) the plasma concentration curves of albendazole and its PEG6000/Poloxamer188 solid dispersion. b) the plasma concentration curves of albendazole and its PVP P30/Poloxamer188 solid dispersion. PVP: PVP K30; ABZ: albendazole; P188: Poloxamer188 (*n* = 3).

**Table 1:** Pharmacokinetic parameters of the administration of albendazole and its solid dispersions to mice. PVP: PVP K30; P188: Poloxamer188.

PK parameters	Unit	Albendazole	Albendazole-PEG/P188-SD	Albendazole-PVP/P188-SD
T_1/2_	h	4.48±3.38	3.83±2.81	4.49±3.76
T_max_	h	0.25±0	0.25±0	0.25±0
C_max_	ng/mL	15.07±3.3	176.52±31	202.54±28
AUC_last_	h*ng/mL	19.82±6.9	108.73±21	116.85±16
AUC_inf_	h*ng/mL	21.98±7.6	110.82±20	119.79±15
AUC__%Extrap_obs_	%	9.60±5.4	2.02±1.31	2.51±1.54
MRT_inf_obs_	h	4.03±2.25	1.39±0.49	1.47±0.62
AUC_last_/D	h*mg/mL	1.98±0.69	10.87±2.1	11.69±1.6

**Fig. 7:**
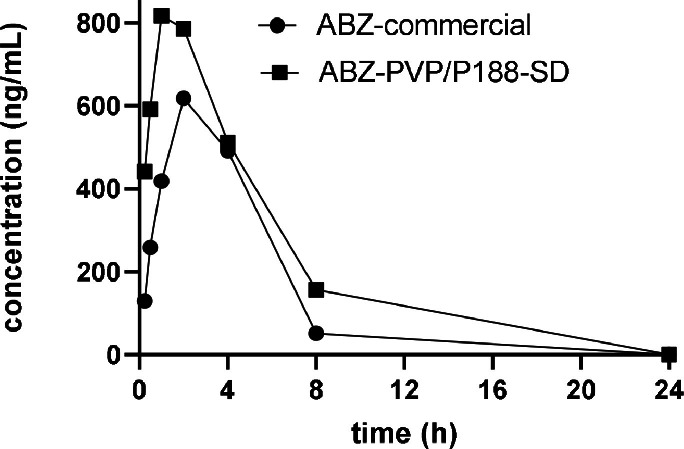
Comparison of the plasma concentrations of Albendazole-PVP/P188 solid dispersion and commercial albendazole tablet. SD: solid dispersion (*n* = 3).

In the subsequent analysis, we conducted a comparison of the plasma exposure between albendazole solid dispersion and a commercially available albendazole tablet following the administration of 50 mg/kg to rats (refer to Fig. 7 and Table 2). The Cmax for the albendazole commercial tablet registered at 630.31±179 ng/mL, while the albendazole PVP K30/Poloxamer 188 solid dispersion exhibited an increase to 803±258 ng/mL. The AUC was calculated through the mean of three rats’ individual AUC plus and minor standard deviation. Furthermore, the AUC for the albendazole commercial tablet amounted to 3365±361 ng*h/mL, whereas the albendazole PVP K30/Poloxamer 188 solid dispersion yielded a higher value of 5344±2182 ng*h/mL, signifying a notable 1.64-fold increase. This observation underscores the significant enhancement in bioavailability and efficacy of albendazole attributed to the use of PVP K30/Poloxamer 188 solid dispersion (Fig. 4,Fig. 6,Table 1, Scheme 1).

**Table 2:** Pharmacokinetic parameters of the administration of albendazole-PVP/P188 solid dispersion and commercial albendazole tablet.

PK parameters	Unit	Albendazole-PVP/P188-SD	Albendazole-commercial
T_1/2_	h	1.98±0.06	2.07±0.15
T_max_	h	1.67±0.58	3.33±1.15
C_max_	ng/mL	803±258	630±179
AUC_last_	h*ng/mL	5275±2290	3364±361
AUC_inf_	h*ng/mL	5344±2182	3365±361
AUC_%Extrap-obs_	%	2.26±3.86	0.0426±0.0235
MRT_inf_obs_	h	4.08±0.54	3.69±0.37
AUC_last_/D	h*mg/mL	106±46	67.3 ± 7.2

## 4 Conclusion

The albendazole solid dispersion was successfully prepared using the solvent method with PEG6000/Poloxamer 188 and PVP K30/Poloxamer 188 as carriers, respectively. Characterization through PXRD, SDC, and SEM confirmed the transformation of albendazole from a crystalline to an amorphous state. Dissolution studies demonstrated a significant increase in the dissolution rate of albendazole solid dispersion, surpassing that of commercial tablets. In vivo pharmacokinetics further indicated a notable enhancement in absorption and bioavailability with the albendazole solid dispersion. In conclusion, the novel solvent method employed for the preparation of albendazole solid dispersion proves effective, offering a straightforward means to achieve consistent amplification in manufacturing.

## CRediT authorship contribution statement

**Ming-Jie Han:** Writing – review & editing, Writing – original draft, Project administration, Methodology, Investigation, Conceptualization. **Zhiyang Zack Zou:** Writing – review & editing, Supervision, Funding acquisition, Conceptualization.

## Declaration of competing interest

The authors declare no conflict of interest.

## Data Availability

All the data was available online.
